# Exploring participants’ experiences of Play to Lead, a leadership development program for adolescent girls in sport

**DOI:** 10.3389/fspor.2025.1727421

**Published:** 2026-01-08

**Authors:** Morgan Rogers, Cari Din, Penny Werthner

**Affiliations:** Faculty of Kinesiology, University of Calgary, Calgary, AB, Canada

**Keywords:** coach support, experiential learning, girls in sport, leadership development, women in sport

## Abstract

**Introduction:**

Despite girls’ and women's increased participation in sport, women remain underrepresented in leadership positions in sport. One of the many strategies that has been proposed to address the dearth of women sport leaders is to begin leadership development in adolescence. The purpose of the present paper was to explore the experiences of participants in Play to Lead, a leadership development program for adolescent girls in sport.

**Methods:**

Interviews and focus groups were conducted with 18 girls and 20 coaches who participated in the program, as well as six Jumpstart staff. Data were analyzed inductively using reflexive thematic analysis.

**Results:**

Based on the interviews and focus groups with the girls, their coaches, and staff, five themes were developed that illustrate how the participants experienced the program: (a) providing both structure and choice for the experiential learning challenges, (b) appreciating the investment in girls’ leadership development, (c) increasing social connection throughout the program, (d) tailoring the program to each community, and (e) coaches supporting the girls’ leadership development. The present results highlight the girls’ generally positive experiences of the program, several suggestions for adjustments to the program based on the girls’ and coaches’ feedback, the benefit of opportunities to practice leadership, and the value of including coaches to support the learning environment.

**Conclusion:**

The present research highlights the relevance of adopting an experiential learning approach in a leadership development program for adolescent girls that provides structure, choice, and opportunities to practice leadership.

## 1 Introduction

Over the last few decades, there has been considerable research that has highlighted the underrepresentation of women in leadership positions in all industries, including sport (1–4). Much of the research has explored the barriers and challenges that continue to exist for women, such as gender bias, a male dominated work environment, pay inequities, and informal hiring networks (2, 4–6). However, there has also been research exploring women, who despite these continuing challenges, have risen to senior leadership positions (7, 8). Further, there is significant research examining ways to advance women in leadership positions within and beyond sport, through mentorship, sponsorship, women's only leadership development and, at an organizational level, through policy development and hiring practices (9–12).

Women's only leadership development programs have been implemented and researched in higher education [e.g., (13)], business [e.g., (14)], and sport [e.g., (15–17)]. Ely et al. (14) and Ibarra et al. (18) argued that women's only spaces are vital as they create opportunities for social support, as well as a safe space to learn, take risks, and make mistakes free from evaluation and judgement. In sport, participants in women's only leadership development programs also valued the opportunity to learn, try new things, make mistakes without fearing failure, and develop supportive relationships with colleagues who share similar experiences (6, 16, 17, 19).

More recently, several scholars have advocated for offering leadership development opportunities to adolescent girls, although much less is known about the best ways to design a program that will be effective for this age group (20, 21). Eva et al. (21) conducted a literature review on leadership development for adolescent girls and noted a number of critical themes, specifically highlighting the importance of peers and key adults supporting girls' leadership development and the need for diverse role models. Building on the literature regarding women's only leadership, the authors also emphasized the importance of providing girls with a safe and supportive environment to practice leadership without fear of judgement. These findings underscore the importance of developing girls’ confidence in their leadership capabilities.

There has also been research in the sport domain on adolescent girls' leadership development and much of the writing noted similar advantages for young girls as has been advocated in the women's leadership development literature, such as diverse role models, mentors, and meaningful practice opportunities [e.g., (20, 22–25)]. Leberman (20) suggested it is critical for girls' leadership development to provide tangible opportunities to practice decision making and create community impact and noted all girls should be provided opportunities to lead, not only those that would typically be defined or chosen as leaders.

Importantly, Gould and Voelker (22) highlighted leadership development needs to be intentionally taught and does not automatically occur as a result of sport participation. In earlier work, Gould and Voelker (26) created a leadership development clinic for high school sport captains, arguing that being a sport captain will not contribute to leadership development without an additional intervention. These authors also suggested leadership development programs shift from lecturing to discussion-based learning and include a program for the coaches to further bolster the athletes' learning after the completion of the clinic. Building on the notion of mentorship and practice, Taylor (27) evaluated a leadership development program for adolescent girls in sport which provided girls with a five-day intensive residential program followed by eight to 10 weeks of leading physical activity sessions with support from a mentor. The program provided the girls with meaningful experiential leadership opportunities and mentorship that contributed to their confidence and the author suggested that this early development of leadership skills might, in the long term, help address the under-representation of women in sport leadership.

When reflecting on leadership development programs, several scholars have written about the importance of adopting an experiential learning approach that creates an opportunity to practice leadership (21, 28–30). The educational theorist John Dewey (31) advocated for the importance of learning through transforming and reconstructing our experiences and yet emphasized that all experiences are not necessarily educative. He noted that an experience alone may not contribute to learning but reflection, and trying to understand and explain a new phenomenon, facilitates learning. Building on this early work, Kolb (32) defined experiential learning as “the process whereby knowledge is created through the transformation of experience” (p. 38) and proposed a model of experiential learning where an individual has an experience, reflects on it, integrates and tests their reflection of the experience, and then actively applies and experiments with their updated frame in relationship to future experiences. This model has been cited extensively by researchers to understand how learning takes place through experience and reflection.

Laritza et al. (30), using Kolb's model, evaluated a leadership development program for women designed to address gender inequity in Spain. The program, which included reflective discussions on topics such as communication, leadership styles, and mentorship, incorporated a personal project that helped the participants practice leadership and develop confidence in their leadership skills. Research by Lawrason et al. (33) and Samuelson et al. (34) supported the need for women and girls to learn leadership through experiential opportunities and confirmed previous research that highlighted a gender gap in leadership development training opportunities, as often women do not receive the same opportunities to practice and build their leadership as their male counterparts.

While this review of literature helps in understanding the value of incorporating experiential learning opportunities within leadership development programs, additional research is needed to build on what is currently known about developing a leadership program for adolescent girls in sport. This paper explores and documents the experiences of participants (girls, coaches, and staff) taking part in Play to Lead, a leadership program for adolescent girls in sport conducted in partnership with Canadian Tire Jumpstart Charities. The program design and subsequent implementation was informed by Kolb's (32) experiential learning theory and evolved based, in part, on participants' feedback and thoughtful reflection by Jumpstart staff and the researchers. The purpose of the present paper was to explore the experiences of participants in Play to Lead, a leadership development program for adolescent girls in sport. As well, the present study contributes to the research literature by articulating how an experiential learning program can be developed for adolescent girls in sport utilizing project-based learning and coach mentors. It also contributes to the research by documenting how the program was improved in response to participant feedback.

## 2 Methods

The present research was informed by an interpretivist worldview, which adopts the belief that reality is subjective, and knowledge is co-created by the researcher and participant (35, 36). Hodge and Sharpe (37) suggested that case studies explore the complexities of a single case from multiple perspectives and that instrumental case studies are used to provide insight into a specific issue. Therefore, the use of an instrumental case study to explore, in-depth, the experiences of adolescent girls in sport taking part in a leadership development program and includes perspectives from the girls, coaches, Jumpstart staff, and the researchers, aligns with an interpretivist worldview (35).

### 2.1 Program description

Play to Lead, a leadership development program and research study conducted in partnership with Canadian Tire Jumpstart Charities (hereafter Jumpstart), has been offered in various locations across Canada beginning in 2022. The program is designed to provide adolescent girls with an opportunity to learn about and practice leadership, develop confidence, and strengthen their social network in and through sport. The design is informed by Kolb's (32) work on experiential learning as well as by the more recent work of Piche et al. (24) and Molina-Lopez et al. (38) who have written about the critical importance of gathering participant feedback and providing adult support for learning. At the time of writing, the program has been delivered across Canada to one cohort in the first year of the program, and two cohorts in the second year. The third year of the program with three cohorts is currently ongoing. The program design evolved with each iteration, based on feedback from the girls and their sport coaches. Each program runs for a period of nine months, using a hybrid delivery model with an in-person kick-off (“the Summit”) followed by online programming and “challenges” with support from the girls' sport coaches and the program team, which consists of Jumpstart staff and the authors (see Table 1).

**Table 1:** Program overview.

Program year	Cohorts	Leadership skills	In-person summit	Online program	Coach involvement
Year one and two.	One cohort (year one) and two cohorts (year two).	Communication. Grit. Authenticity. Problem solving. Leading a group.	Two days. Topics and activities such as a workshop on effective communication, presentations by elite women athletes, and physical activity sessions, such as playing wheelchair basketball.	Monthly 45-minute online sessions discussing leadership skills, and individual experiential learning “challenges” with regular feedback, as well as a brief post-challenge reflection (challenges included conducting an interview, organizing a team practice, and creating an action plan to address an issue in sport.)	Participation in the Summit with several coach-only sessions (topics such as effective communication, adapted physical activity). In year one, monthly coach sessions (e.g., coaching girls, grant funding applications, and mental health). In year two, no monthly sessions.
Year three.	Three cohorts.	Communication. Embodying Values. Navigating conflict. Reflective practice. Peer mentoring. Managing a group. Leadership identity. Resilience.	Three or four days, depending on the cohort. Topics and activities such as communication and conflict management workshops, introducing the personal leadership challenge, presentations by elite women athletes, and physical activity sessions.	One nine-month personal leadership challenge, completed individually or in groups. Four 60-minute online sessions to support the personal leadership challenge. Submission of four personal reflections on successes and challenges, what they were learning, and an action plan to integrate those lessons into their challenge.	Participation in the Summit and coach formal mentorship training. Coaches responsible for supporting athletes’ personal leadership challenges.

In the first two years of the program, the girls attended monthly 45-minute online learning sessions centered on a specific leadership skill (see Table 1) and then completed an associated individual experiential learning “challenge.” The program used the term challenge to indicate an opportunity to practice the skill discussed in each online session. Individual feedback was then provided by the staff on each “challenge” via an online platform.

In the third year of the program, the girls in each cohort participated in an in-person Summit (similar to year one and two) but based on feedback from the earlier cohorts, the monthly challenges were replaced with a personal leadership challenge that ran throughout the nine months of the program. Throughout this third iteration of the program, four 60-minute online sessions were designed to support the work of the personal leadership challenge, including project planning and how to communicate the final outcome.

In each cohort, the girls were prompted to complete brief individual reflections on each of the challenges and submit those written reflections online (i.e., monthly in year one and two; four times in year three) exploring what worked well, what was difficult, what they learned from their successes and challenges, and how they could integrate those lessons into the next part of their program. These regular reflections were a critical component of the program prompting the girls to think more deeply, become comfortable in asking questions when they did not understand, and work toward transferring that learning to other parts of their life.

### 2.2 Participants

The girls and coaches were recruited to participate in the program based on their affiliation with an organization that had previously received community sport funding from Jumpstart. Following the conclusion of each of the first three cohorts, participants were invited to be part of the research. At the time of writing, the third year of the program was still ongoing, therefore the participant data focuses on years one and two (three cohorts) while staff data considers the first two years of the program as well as changes to the design for the third year. Study participants included a convenience sample of 18 coaches (nine in year one/cohort one; two in year two/cohort two; seven in year two/cohort three), 20 girls aged 14 to 18 (13 in year one/cohort one; five in year two/cohort two; two in year two/cohort three), and six staff from Canadian Tire Jumpstart Charities (36). Following guidance from Sandelowski (39), the sample size is large enough to contain rich and varied data, while small enough to conduct an in-depth analysis. All of the recruited participants completed the research study and there were no withdrawals. The girls and coaches participated in a variety of individual and team sports such as soccer, basketball, field hockey, swimming, and athletics. Participants were located in urban and rural communities in the region of the host cities, which were Toronto, Ontario (one cohort in year one and one cohort in year two) and Edmonton, Alberta (one cohort in year two). For the purpose of this study, girls, coaches, and staff were assigned a code to ensure confidentiality.

### 2.3 Data collection and analysis

Prior to beginning data collection, the study received ethical approval from the University of Calgary Conjoint Health Research Ethics Board (CHREB) and all participants provided informed consent. In line with guidance from the university ethics board, study participants aged 14 to 17 were considered mature minors and were assessed for ability to provide full consent using the following criteria: the minor has the ability to reason and understand the benefits and potential risks associated with participation, the minor has full information about the benefits and potential risks associated with participation, and the minor feels no compulsion to participate (40).

The first author is considered a participant-observer in Play to Lead*,* based on her collaboration with Jumpstart staff on program design and implementation as well as attendance at all the Summits and online sessions over the course of a three-year period from 2022 to 2025 (41). Following the conclusion of the three cohorts in the first two years, the girls were invited to participate in a focus group and their coaches were invited to participate in an interview to discuss their role in the program (i.e., as a girl participant or a coach), share feedback based on their experiences in the program, and explore design considerations for future cohorts. Throughout the three years, as the program design and implementation evolved, Jumpstart staff were also invited to participate in an interview with the first author. Interview questions for the girls included: “Tell me about your experience participating in the program”. “What have you learned in the program?” “What was difficult?” Questions for the coaches included: “Describe your role as a coach in the program” “How did you work with your athletes in the program?” “Do you feel the athlete you coached has learned about leadership throughout the course of this program? If yes, what might that be? And if no, why not? What stood in the way?” Questions for the staff included: “How has the program evolved since you started working with the program?” “What are the strengths of the program, and what could be improved?” “How will the program continue to evolve moving forward?”

Data were collected and using Braun and Clarke's (42, 43) reflexive thematic analysis (RTA), an approach that views researcher subjectivity and connection to programs as an analytic asset. Therefore, the first author's experience as a participant-observer [i.e., someone who participates but primarily observes from the periphery (41)], in the Play to Lead program strengthened the data collection, analysis, and interpretation. Indeed, Braun and Clarke (44) argued “themes cannot exist separately from the researcher—they are *generated* by the researcher through data engagement mediated by all that they bring to this process (e.g., their research values, skills, experience and training)” (p. 39). Braun and Clarke (43) outlined six phases for reflexive thematic analysis: familiarization with data, systematic coding, generating themes, reviewing and naming themes, finalizing themes, and creating the report.

The first author familiarized herself with the data by transcribing the interviews and focus groups and taking notes on the transcripts and her field notes, coding the data, and creating initial themes. The first author reread and reflected on the transcripts multiple times to scrutinize and refine the themes she noticed and developed as the program evolved over the course of the three years, which Braun and Clarke (42) suggested is critical in the data interpretation and analysis process. Further, this process of concurrently collecting and analysing the data influenced changes to the program design, in addition to the first author's analysis and interpretation of the data and the creating of draft themes. She then shared and discussed draft themes with her co-authors, who provided an outside perspective and served as critical friends to scrutinize, deepen, and clarify the interpretations and subsequent themes, which were discussed and further developed over multiple iterations (42). For example, an early theme described the value of including the coaches in the program. However, discussions between the three researchers and conversations with Jumpstart staff focused on iterative improvement of the program and this theme was critiqued, challenged, and ultimately modified. This theme now illustrates greater interpretation and nuance regarding coach involvement, and that while it is generally positive element of the program, it is not without its challenges.

### 2.4 Rigor

Given her role as a participant-observer, it was critical the first author practiced reflexivity, which refers to reflecting on one's position and how it may be impacting the research process (45). In the present research, the first author engaged in monthly reflexive conversations with her graduate supervisors and co-authors, who served as what Smith and McGannon (46) refer to as “critical friends” (p. 113). The co-authors provided an arm's length perspective on the program, asked critical questions and pushed the first author to think deeply about her emotions, dual role as a researcher and practitioner in the Play to Lead program, and relationships developed with study participants (47). For example, after one of the reflexive conversations between authors, the first author noted in her journal “because I am close to the program and care a great deal about the people involved, I am struggling with the critique”. Therefore, the process of monthly reflexive conversations between authors and personal reflection enabled the first author to critically examine the data more deeply. Finally, a thick description, which relies heavily on the use of quotes, was used to allow participants and staff to narrate their experiences and reflections on the program in their own words (36).

## 3 Results

### 3.1 Section one: five themes

#### 3.1.1 Providing both structure and choice for experiential learning challenges

Within each iteration of the program, the girls were responsible for completing a variety of challenges related to the leadership skills that were discussed in each of the online sessions, such as how to effectively communicate, develop resilience, build their team culture, navigate the difficulties they might face in one of the challenges, and plan a project. Many of the girls spoke about how they appreciated having structure in terms of suggested topics, expectations, and timelines, as well as flexibility in how they approached and completed each of their challenges. Girl 3 stated “the challenges were simple to understand, you broke it down so it wasn’t complicated. You know those school projects where you have no idea? I thought these were clear and simple to complete”. Girl 9 noted she would have preferred even greater direction:

I liked the open part, but for my personality I like being told exactly what I need to do and not just ‘here you go’ and that's what led me to having questions in the online sessions, because I wanted to be sure I was on the right track.

In terms of appreciating the choice and flexibility once the challenge was outlined, Girl 1 noted:

The flexibility was great because some months I was busier than others, so it was nice having a choice how to structure my work. When I had more time and energy I could put more creativity into the challenge, and the months where I didn't have as much time I could still get something done that was really good. In the months where I had more time, I could make a video and edit it, and months where I didn't have time, I wrote something.

Girl 10 said, “I liked how you could make it whatever you wanted it to be, they would tell you what the challenge is but you could have as much fun with it as you wanted”. Girl 4 remarked “school is more one-sided when you’re learning, whereas in this program you could share your own ideas and share who you are”.

Certainly, the opportunity to choose how they would complete each challenge was an opportunity for the girls to develop their leadership. As Girl 14 said, “I think completing the challenges has helped improve my leadership because they make me reflect on what I’m doing with my project and where I’m going with it”. Building on that, Girl 20 added “as well as having the power of your own creativity and imagination to make it [the challenge] all about who you are and reflect your personality, you still contribute to your community”. Several of the coaches noted how they were working to support the girls and yet, at the same time, promote taking responsibility for completing the challenges. Coach 12 stated, “the athletes had a group chat going and picked up a bit of leadership from that and captained their own ship”.

#### 3.1.2 Appreciating the investment in girls' leadership development

Throughout the three years, there was considerable feedback from many of the girls about their appreciation for the program and the support they received throughout the nine months. Girl 10 stated “I personally am very grateful for them [program staff]. I loved the whole community. And the ‘hey, we’re here to talk’ if I had any questions”. Girl 9 stated the support from the program communicated to her what she was doing was meaningful: “the staff were so generous, and I really felt like they appreciated us and the work we’re about to do and that reassurance that this is actually helping us develop and can lead to something”.

In addition to this support, the program staff reviewed each of the girls' challenges and provided feedback on a regular basis (monthly in the first two years, and four times in the third year). The girls recognized the time and effort, and Girl 16 expressed: “after each challenge, after a leader had reviewed it, they would comment on it and tell you what you did well and some things like “maybe next time add this” and I felt that really helped me learn”.

The coaches also commented on the importance of the program and stated that valuing and supporting the girls was critical at this point in their lives as they developed their identities as individuals as well as athletes and young leaders. Coach 16 noted:

The Play to Lead staff were really cognizant of ways to keep the girls motivated and at the graduation, every single girl had something highlighted about how she contributed. She got off that call and was on such a high. She's like “they were really listening”. That's so important. Just hearing that, wow, she felt special. You did a really good job of making every single girl that was involved feel special enough to want them to keep pursuing athletics.

Coach 15 elaborated on that sentiment, stating:

Especially for young women, the ability to actually feel heard. To feel heard and be seen in their experience was so powerful and so empowering for these girls. You can’t buy an experience like that, and this will stay with these young women forever.

#### 3.1.3 Increasing social connection throughout the program

In considering ways the program could be improved, the participants, both the girls and their coaches, expressed a desire for increased social interaction with one another. Throughout the three years, it was consistently shared that the in-person Summit was a great opportunity to interact and build relationships with fellow program participants. Coach 1 shared the following reflection from observing the athlete she worked with at the Summit:

Seeing her shift from day one to the next day, being around other athletes who are leaders in their own sport. For her to interact with other athletes has really brought her out of her shell. And it's not about competition. It's about encouraging everybody around you. As women, typically everything's a competition, we're trying to compete for everything, whereas you recognize there's space here for me and there's also a space for you to be our best selves. I want to help you grow, you want to help me grow, so just watching her recognize that was pretty cool.

The girls expressed a desire for increased opportunities to continue to develop their relationships following the Summit. Girl 9 remarked: “at the Summit it felt like a team but then in the online sessions it felt like I was not part of a group program and instead part of an individual program”. As a result of this feedback, the program team increased the length of the online sessions to allow time for informal social connection between participants.

Further, many of the girls suggested it would be beneficial to create opportunities to collaborate with others on the challenges, with Girl 17 noting:

Having a challenge where you're paired up with one or two people, and you have to complete the challenge with them would be great because we worked together at the Summit and then we got to see each other in the online sessions, but you were doing your own thing. So having a challenge where you work with some of the other athletes would be nice.

Girl 18 built on this sentiment, noting she was grateful to have a teammate in the program to provide a social connection: “it was definitely nice having a teammate in the program because it made you feel like you weren’t alone”. In response to this feedback, the program offered the girls the opportunity to work in groups in the third year of the program.

Finally, both the girls and their coaches stated that they would like support from the program staff in staying in contact with their fellow participants following the conclusion of the program. Girl 1 said:

Having a concrete way to keep the connections going would be nice because I met some really cool people but I'm just not sure how to keep this going … if we as a group want to keep supporting initiatives for girls' sports, such as going to a female game. For example, there's a summer league for professional women's basketball called HoopQueens and it would be great to be able to say ‘hey, this is super cool, does anyone want to come with me and watch and support female basketball in Toronto?’

Coach 14 expressed a similar sentiment:

I'm hoping that there will be continued connection between the girls and with the other coaches over the years to come because too often we do something and it impacts us and then what? A continuing connection could add value to the process as we try to build a community.

#### 3.1.4 Tailoring the program to each community

In the first year, the program was offered in one city and, as the program expanded, the number of cites expanded to two in year two and three in year three. Based on the feedback provided by the girls and their coaches following the second year of the program, in particular related to the more popular sessions and facilitators in each region, the program staff discussed the importance of more carefully considering what might be relevant for each community. For example, Staff 6, after reviewing the feedback, reflected on the need to tailor program offerings in each community: “I think we saw the difference in the workshops people really liked in each city. The workshop on communication was rated highly in one city by the girls, but quite low in another city”. Building on that*,* Staff 1 noted:

We took the same approach from the first location and applied that in the second location, and it became very clear that if we're meeting a community need, we need to listen more carefully to each community. The program's overall desired outcomes will be the same, but the program delivery might not look the same in each location. I think it was a learning for us that the approach of taking the same version of the program location to location doesn't work.

In part, based on this feedback and reflection, the program staff worked with local community groups as part of the design process in the third year, to ensure the program offerings were aligned with the needs and interests of the various communities. Staff 6 stated:

We know that designing and programming solely in the head office isn't necessarily the best way to design programs anymore. So it has really shifted from a bunch of adults in an office designing a program and then doing that program the same way from one city to another, to a design approach with input from community partners, as well as from the youth that were previously involved in the program.

As a result of these conversations, the program design for the third year was altered considerably with the program staff tailoring the delivery to ensure the program was aligned with regional needs. For example, the program staff worked with indigenous facilitators for the cohort in an indigenous community and delivered the program in French for the cohort in the province of Quebec.

#### 3.1.5 Coaches supporting the girls' leadership development

In each iteration of the program, the coaches attended the in-person Summit and participated in their own educational programming to support their work in the program (e.g., topics such as athlete mental health, coaching girls, applying for grant funding). They were asked to support the girls as they completed their challenges and several of the coaches noted they also worked to create additional leadership opportunities beyond the program, such as mentoring the girls to begin to think about a coaching role within their community.

Several of the girls noted it was their coach who first encouraged them to participate in Play to Lead and spoke about appreciating their support. As Girl 5 noted: “it was definitely helpful to have my coach in the program and go through it with her and just be able to have her understand what's happening or run ideas past her”.

Over the course of the three years, the program staff continued to seek feedback and reflect on how best to involve the coaches to maximize their role and support. As Staff 4 noted:

The role of the coaches is critical and speaks to the sustainability of the program because ideally, if we can keep quality coaches in the system, they're going be able to support generations of future female leaders. The more we can improve their knowledge, skills, and capacity, the more we've got an ability to sustain that development of future female leaders and sport in that geographical area.

However, there was also feedback from the coaches that noted a desire to be more involved and have greater role clarity. As Coach 1 noted:

I would have liked to do more with the athlete … to me, the whole point of this program is to encourage these girls to stay in their sport so having them shadow and be a little more involved with us, I think that's what I was hoping for.

Coach 12 expressed a similar sentiment, noting: “I was not as involved as I expected to be, but I had 16-17-18-year-olds who were independent. If I had only 14-year-olds, I would require more support and clarity from the program”.

In addition to delineating a clear purpose and role for the coaches, an additional challenge the program staff had to navigate was managing the coaches' differing availability and motivation for engaging in the program. Many coaches were very involved in supporting the athletes and took on a significant role, while others noted they were volunteer coaches with limited time and capacity to dedicate to the program. Coach 17 remarked: “I have two jobs, so I tried my best to follow along and check in with the girls, but it would have been easier if the coaches had check-ins on what we should be doing”.

Over the course of the iterations of the program, the staff began to understand the issues facing the coaches, with Staff 6 reflecting:

The coaches did not really understand their role because we didn't set them up for success. We had some that took a lot of initiative, and I think contributed to the success that the girls had and others were overcapacity or didn't understand what was required and I think that's probably to the detriment of the girls and the program. I think we need to structure the role of the coach in a more concrete way.

### 3.2 Section two: a story of leadership: a community initiative

In the first year of the program, one of the girls (Girl 2) worked closely with her coach to turn an idea for increasing opportunities for girls to play the sport of rugby into one of her monthly program challenges. She then transformed her challenge into a five-week initiative, with support from her coach, which has now been hosted for three consecutive years. She spoke about some of the conversations she had with her coach around building the initiative, as well as some of her own reflections:

I brainstormed this plan with my coach as I was submitting it for my challenge in the program. I was like “this is something that I could really carry out in my community”. I talked to my coach, and he had already been thinking of something like this, so it was the perfect opportunity to really make this vision come to life. We met a few times. We wanted all girls to be included and then we looked at the barriers for girls in rugby. We brainstormed “what is the purpose of this initiative?” and really wanted to build connections and make friends. Implementing the rugby initiative has been great and it's important to me because I see my vision coming to life and it's a bunch of girls playing rugby and learning together.

She also reflected upon how participating in Play to Lead contributed to her ability to develop her own initiative for girls in rugby:

After participating in the program, I knew that I had the leadership skills and I had done those different challenges throughout the months that I needed for this initiative, so I knew this was something that I could take on. To have the confidence the program built in me to be able to say, “hey, this is a great opportunity, I’m gonna jump on this”.

As well, the athlete spoke to the importance of the experiential learning created by the monthly challenges:

The challenges really gave you an opportunity to apply this in real life, because I feel that sometimes, even if you talk about something, it's not until you really carry it out that you're able to learn. And with this rugby initiative, not only does it make an impact on the community, but it allows me to build my leadership skills in the field as well.

Her coach (Coach 2) spoke of supporting this initiative:

We talked about the mission, vision, and values of what we should do and she drafted it up and then pitched it to Jumpstart for funding. She then pitched it to our club coaches. There must have been 60 or 70 people and she addressed the whole group. She also said to me “okay there's certain criteria that we must meet for our funding, who's the person that I need to communicate with at the club that is head of marketing and social networking, how do you want me to do this? What's the best strategy? Where do we go next?” and I'm sitting there like “oh my gosh, I can’t believe you have this all together”. So, yeah, that's one example where she just started digging in.

The program staff reflected on the success of this rugby initiative, both in terms of achieving a wider community impact as well as providing this adolescent girl with an opportunity to actively practice leadership. This initiative influenced a subsequent shift in program design for year three of the program, moving from a series of discrete monthly challenges to a single challenge conducted over the nine months.

## 4 Discussion

The purpose of the present paper was to explore the experiences of participants in Play to Lead, a leadership development program for adolescent girls in sport. Jumpstart staff and the first author designed and facilitated the program and collected feedback from the girls and their sport coaches from the first three cohorts. Based on the interviews and focus groups with the girls and their coaches, as well as the staff, five themes were developed that illustrate how the participants experienced the program: (a) providing both structure and choice for the experiential learning challenges, (b) appreciating the investment in girls' leadership development, (c) increasing social connection throughout the program, (d) tailoring the program to each community, and (e) coaches supporting the girls' leadership development. In addition to the themes, one participant's story is presented, which illuminates how she and her coach took one of the monthly challenges and created a unique initiative for girls and rugby in their community, providing an in-depth illustration of one participant's practical application of her leadership skills. The present results also highlight the girls' generally positive experiences of the program, several suggestions for adjustments to the program based on the girls' and coaches' feedback, the benefit of opportunities to practice leadership, and the value of including coaches to support the learning environment.

With the understanding that leadership development needs to be intentionally facilitated, Play to Lead is designed to actively foster leadership development for adolescent girls in sport (20, 22). A critical component of the design is the use of experiential learning theory with staff providing structure and direction while ensuring the girls are able to choose how they complete their challenges. These findings reinforce the value of Kolb's (32) model of experiential learning as the girls and coaches noted that the challenge-based design of the program supported the girls' development of leadership skills. It is perhaps not surprising that the girls valued and benefitted from having the opportunity to practice leadership, as recent work by Laritza et al. (30) and Molina-Lopez et al. (38) also found value in similarly designed programs. It is also important to note that Lawrason et al. (33) has suggested that opportunities to practice leadership are particularly critical for girls and women since they often receive fewer leadership opportunities than their male counterparts. The findings also reinforce the research that speaks to the benefit of a girls-only environment that creates a supportive space for girls to learn and grow as future leaders (6, 16, 30, 38).

A strength of the design of the Play to Lead program is the integration of regular feedback from the participants, and as a result, staff have been able to adjust the content and the challenges to meet the needs of the girls and their coaches. This finding supports the work of Piche et al. (24) who noted that such an approach increases engagement and enables participants to take ownership for their learning as they actively practice leadership. Eva et al. (21) argued “there is compelling qualitative evidence that by engaging in co-construction, adolescent girls have demonstrated to themselves that they are leaders” (p. 9). Indeed, the girls and coaches in the present research shared that the process of being both encouraged to provide feedback and see that their comments and suggestions were being considered contributed to their sense of being valued as potential young leaders in their sport community.

Building on the notion of investing in young girls as leaders, Leberman (20) suggested that it is important to provide leadership development opportunities to girls from a variety of backgrounds and not only to those who would typically be identified as a leader. The most recent iteration of the Canadian Sport Policy has highlighted the need to increase access to sport programs for those living in rural and remote communities (48) and Play to Lead, by adopting a hybrid approach with one in-person gathering followed by online sessions, was able to reach participants throughout Canada, including those who reside in rural communities.

In reflecting on the hybrid design, online programming is a promising approach that can address financial and geographical constraints, and several scholars have argued it is an accessible approach to sport programming that can be adopted within and beyond the Canadian context (49–51). The girls in the present study appreciated the flexibility afforded by online programming, which supported the girls' school and sport schedules, with one of the girls noting “you can do it anywhere even if you’re busy, sometimes I attend the [Play to Lead] sessions from [sport] practice”. It is important to note that while there are many advantages of online programming, there are also limitations to this approach as the present study also found that several of the participants indicated they would have liked more social connection. Crucially, in response to that feedback, program staff adjusted the third year to add time for socializing within the online sessions. Nevertheless, it will always be important to consider both the advantages and disadvantages when designing a hybrid learning program.

A unique component of the Play to Lead program was the inclusion of the girls' sport coaches. The program staff understood the critical role coaches can play in young girls who participate in sport. Many of the young girls spoke of how their coaches encouraged them to apply for the program and served as a valuable sounding board and significant support once they were working on their leadership challenges. Several of the coaches also shared that, when they were able, they sought out additional leadership opportunities for the girls to engage in beyond the program. These findings support the research of Fraser-Thomas et al. (52) that has highlighted coaches are uniquely positioned to support and create leadership opportunities due, in large part, to their prolonged interaction with athletes in their sport setting. However, scholars have also noted that to do this effectively, it is important to provide coaches with adequate education and role clarity to ensure they are well prepared to provide support (53–55). In the present research, the coaches expressed appreciation for the professional development sessions they were offered. However, there was some confusion about the role of the coach and at times it was difficult to determine how best to utilize the expertise of the coaches. In some cases, the coaches themselves were unclear about their role and in other cases, they simply did not have enough time to contribute substantially, as they had other full-time employment, which is aligned with a recent report which highlighted that in parts of Canada, 76% of coaches are unpaid volunteers (56). While much has been written about the positive impact coaches can have on an athlete's development through developing supportive relationships and providing feedback, much less has been written about the time and financial barriers community sport coaches face, and the potential impact this might have on program outcomes (54, 57). As future iterations of Play to Lead continue, it will be important to clearly delineate the coaches' role and ensure there are adequate resources provided to support the coaches. Moving beyond the program, addressing systemic barriers for coaches, such as time and financial constraints, to maximize the impact they can have on youth development in sport is a priority area in the new Canadian Sport Policy and an area where further research is needed (48).

The story of one of the participants in year one, building a rugby program for young girls in her community, points to another strength of the Play to Lead program's design and its process of implementation. The program worked to create leadership content, time for questions and reflection, and concrete challenges to practice the skills of leadership. In this story, one of the girls and her coach applied what they experienced in the sessions to go beyond what was asked and develop a rugby program for young girls in their community that still runs to this day. This is a remarkable achievement due in large part to the Play to Lead program. As the program has evolved from monthly challenges to a personal leadership challenge over the course of the nine-month program, additional examples of leadership projects and community impact are beginning to emerge.

### 4.1 Strengths of the study

In summary, the present research explores the experiences of the participants taking part in a leadership development program for adolescent girls in sport. While the Play to Lead program continues to be refined with each iteration, it is important to highlight three notable strengths, with the first being the willingness of the program staff to listen to the participants on an ongoing basis and importantly, adjust the program accordingly. Second, the program developed an environment that enabled one of the young girls, with help from her coach, to create a remarkable initiative in her community for young girls in the sport of rugby. Third, the integration of the girls' sport coaches into the program, while not without its challenges, provided significant support for the girls' leadership development. While parts of the program will continue to evolve and improve, the Play to Lead program is a promising avenue for change for girls in sport in Canada.

### 4.2 Limitations and future directions

A potential limitation of the present research is that the first author was a member of the program team and, as a result, the participants, in the focus groups and interviews, may have felt a need to emphasize positive aspects of the program. However, this limitation is addressed, at least in part, by providing a thick description of the participants' voices in how they experienced the program and their suggestions for adjustments for future programming. A second potential limitation of the current Play to Lead program was the turnover of program staff throughout the three years and, as a result, some of the feedback took longer to address. However, as new staff joined Jumpstart, they were eager to review and address ways to improve the program. Finally, the convenience sample is a limitation of the present research as those who were more engaged in the program and had positive experiences were potentially more likely to participate in the research, while divergent perspectives may have been missed (35). Future research could explore the long-term impact on participants' leadership behaviours, as well as their retention in sport as participants and leaders as the program continues.

## 5 Conclusion

This research study explores the experiences of the participants in Play to Lead, a leadership development program for adolescent girls in sport, conducted in partnership with Canadian Tire Jumpstart Charities. Adopting an experiential learning approach that provides participants with both structure and choice and actively listening to and tailoring the program to participant needs based on their feedback were strengths of the program. The inclusion of the girls’ sport coaches was a unique feature of Play to Lead, and while not without its problems, particularly in terms of role clarity, it contributed positively to many of the girls' learning experiences. Finally, the community initiative by one of the participants, creating an opportunity for many young girls to play rugby, certainly confirms that the program design and implementation created an environment where participants could take their leadership skills beyond the program and positively impact their communities.

Based on the findings, it is recommended that leadership development programs for adolescent girls in sport create meaningful opportunities for experiential learning through providing participants with choice and autonomy over their learning. It is also recommended that facilitating opportunities for social connection will aid in developing confident young leaders and will be instrumental in ensuring girls stay involved in both playing and leading in sport. Further, programs targeting adolescent girls in sport are encouraged to include participant voices in the program design, either through soliciting and acting on feedback on an ongoing basis, or through a co-creation process in the initial program design.

## Data Availability

The datasets presented in this article are not readily available due to privacy concerns. Requests to access the datasets should be directed to morgan.rogers@ucalgary.ca.
